# Multiscale Pore Structure Characteristics and Crack Propagation Behavior of Coal Samples from High Gas Seam

**DOI:** 10.3390/ma15134500

**Published:** 2022-06-26

**Authors:** Jie Zhu, Tangsha Shao, Guiyou Li, Yuhang Yang, Zhen Chen, Tianxiang Lan, Jinge Wang, Yuhan Zhao, Shuangqing Liu

**Affiliations:** School of Mechanics & Civil Engineering, China University of Mining & Technology (Beijing), Beijing 100083, China; shaots2021@163.com (T.S.); mh5451899@126.com (G.L.); yyhnina@163.com (Y.Y.); zhenchen202205@126.com (Z.C.); lantianxiang111@163.com (T.L.); cumtbwjg@163.com (J.W.); zhaoanintendo@163.com (Y.Z.); lsq6396@163.com (S.L.)

**Keywords:** pore size distributions (PSDs), crack propagation, surface fractal dimension, mercury injection porosimetry (MIP), three-point bending, high gas coal seam

## Abstract

Investigation on the pore-fracture features and crack propagation behavior of coal is necessary to prevent coal mine disasters. The pore structure features of coal samples taken from high gas seam were obtained by mercury injection porosimetry (MIP) and gas adsorption methods. The process of deformation and failure for coal samples under three-point bending conditions were obtained. The results demonstrate that the adsorption pores with diameter less than 100 nm are the most developed and their surfaces are the roughest (the average surface fractal dimension *D*_s_ is 2.933). The surface of micro-cracks is smoother (*D*_s_ is 2.481), which is conducive to gas seepage. It may be the explanation for that 14-3# coal seam is a high gas seam, while there was almost no gas outburst accident so far. At the initial stage of crack propagation, the main crack on the coal sample expanded along the direction of the natural cracks. In the process of crack propagation, the surface fractal dimension of the main crack increased, suggesting that the bending degree of the main crack enhanced. The brittle characteristics of coal samples can be reflected by the ratio of the dissipated energy to the accumulated energy.

## 1. Introduction

It is known that coal is a quasi-brittle porous medium with a pretty low tensile strength compared to its compressive strength, which is prone to cracking [1]. The extension and coalescence of micro cracks and multiscale pore structure have significant influence on the mechanical properties and fracture toughness of coal seams [2,3,4]. Research on multiscale pore structure characteristics and micro-crack propagation is necessary.

Combined with classifications of pores proposed by Hodott [5] and the International Union of Pure and Applied Chemistry (IUPAC) [6], the pores are divided into the super micropore (*d* < 2 nm), micropore (2 nm < *d* < 10 nm), transition pore (10 nm < *d* < 100 nm), meso pore (100 nm < *d* < 1 μm), macro pore (1 μm < *d* < 10 μm) and micro fracture (*d* > 10 μm), where *d* is pore diameter. Among them, pores with diameter less than 100 nm are treated as adsorption pores, and that between 100 nm and 10 μm are regarded as seepage pores [7]. There are various methods for investigating the pore characteristics of porous medium, such as gas adsorption, mercury injection porosimetry (MIP), acoustic emission (AE), computed tomography (CT), scanning electron microscope (SEM) [8,9,10,11,12,13]. Among them, MIP is usually used to describe the feature of pores with the size from 3 nm to 300 μm. However, when the mercury injection pressure reaches a certain value, the solid matrix is compressed, resulting in a larger mercury injection volume [14,15]. Low-temperature nitrogen gas adsorption (LT-NGA) and low-temperature carbon dioxide adsorption (LT-CA) methods can reveal the characteristics of micropores and transport pores (less than 300 nm). Admittedly, gas adsorbed in coal samples, will result in matrix expansion effects, and quadruple moment [16,17]. Some scholars have pointed out that using gas adsorption data to modify MIP results can obtain more accurate pore volume [12,13,15]. 

The pore size distributions (PSDs) of coal specimens are characterized by randomness and self-similarity [18]. Fractal dimension is widely applied to quantitatively evaluate the spatial heterogeneity and the surface roughness of porous media. The larger the fractal dimension, the rougher the surface [19,20]. The popular fractal methods are the Friesen and Mikula model [21], Neimark dimension [22], FHH (Frenkel–Halsey–Hill) model, BET model, Langmuir model, Zhang and Li model [23,24], multifractal model, generalized fractal model [13], and box counting model [25], etc. However, certain ambiguities remain when the fractal dimensions from different models are compared with one another [26,27]. Based on MIP data, Zhang and Li studied the roughness of different pore size regions of bi- and multi-disperse porous solids [23,24]. Gagnepain and Carmes proposed box counting fractal model which is associated with two-dimensional or three-dimensional images [25].

Tensile strength and fracture toughness are important to investigate fracture properties of brittle materials [8,28,29]. Due to simple operation and easy machining, a three-point bending experiment is always used to measure the fracture toughness of materials. Numerous scholars have studied the effects of sample size [30], incision-to-depth ratio [31,32,33], loading rate [34], temperature [35,36], and bedding angle [37,38] on the fracture parameters of quasi-brittle materials. Agioutantis et al. [8] through the acoustic emission technology detected the crack evolution of marble under three-point bending conditions. Zuo et al. [35,37] was based on a three-point bending experiment to study the tensile strength and fracture toughness of small-scale rock samples under different temperatures and joint conditions, and SEM was applied to observe the initiation and propagation of micro cracks simultaneously, which is of great significance to understand the formation of macroscopic visible cracks. However, there are few studies on the mechanical properties and crack propagation of small-scale coal samples under a three-point bending test.

In this paper, the distributions of the pore and micro fracture for coal samples selected from high gas seam were obtained by MIP, LT-NGA, and LT-CA methods. The surface roughness of pores and micro-cracks with different pore size intervals were characterized by surface fractal theory. Under the three-point-bending condition, the deformation and failure of coal samples were investigated by SEM. At the same time, the curve of load-displacement, the characteristics of crack propagation, and other mechanical properties for the samples, such as tensile strength and fracture toughness, were obtained.

## 2. Samples and Methods

### 2.1. Sample Preparation

**Figure 1:**
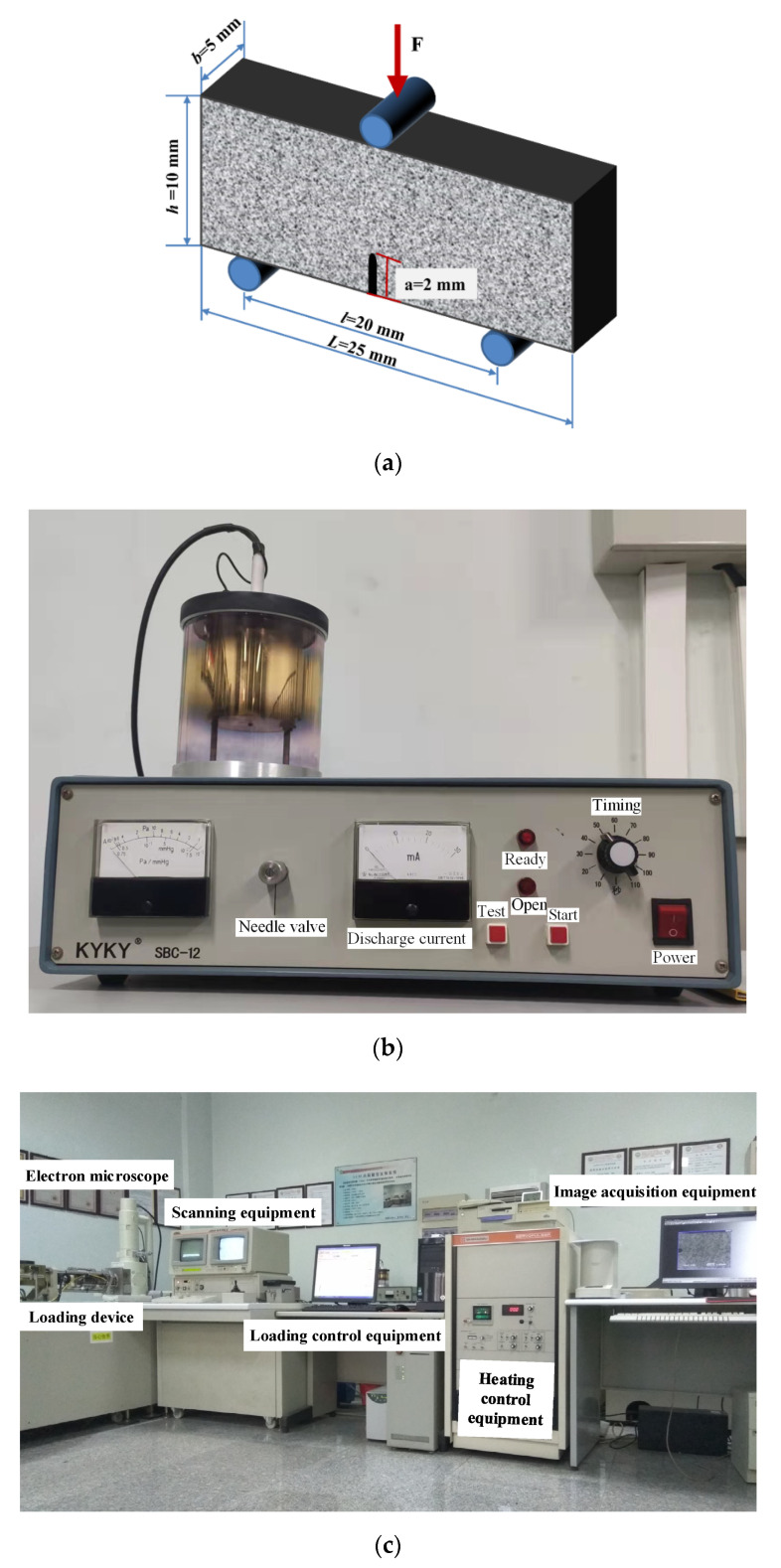
The diagram of coal sample and equipment for three-point bending experiment: (**a**) Diagram of three-point bending specimen; (**b**) Gold-plated equipment; (**c**) SEM fatigue experiment system.

The coal blocks used in the experiments were collected along with the bedding plane from the 14-3# coal seam (high gas mine) of Xinzhouyao (XZY) Coal Mine in Datong City, Shanxi Province, China, with a sampling depth of 313~357 m. The gas emission rate of this coal layer is 32.55 m^3^/t [39]. The collected coal blocks were processed into a cuboid with a length of 25 mm, a width of 10 mm and a thickness of 5 mm, due to the limitation of the loading system and SEM devices. The long side of the coal sample is parallel to the bedding plane. To capture the crack evolution, a U-shaped notch with a length of 2 mm and a width of 0.4 mm was prefabricated on the sample, as shown in Figure 1a. The span distance between two support points was set as 20 mm. The above sample-making scheme was carried out in strict accordance with the method recommended by International Society for Rock Mechanics (ISRM) [40]. The small pieces, remained after making the cuboid samples, were collected for MIP and gas adsorption experiments.

### 2.2. Experimental Equipment and Schemes

The Auto Pore IV 9520 instrument was employed at the MIP experiment at Tsinghua University in China. The sizes of the coal specimens were cubes with a side length of 1 cm. The mercury-coal contact angle was 130°, and the surface tension was 0.485 N/cm. The mercury injection pressure increased to 413.44 MPa, and the pore diameter was calculated by Washburn’s equation [41]. The LT-NGA and LT-CA experiments using power samples with particle size of 0.20–0.25 mm were performed by an ASAP 2460 automatic gas adsorption apparatus at Tsinghua University. The temperatures for LT-NGA and LT-CA experiments were 77 K and 273 K, respectively, which could gain the characteristics of pores with the sizes ranging from 2 nm to 300 nm and less than 2 nm, respectively.

The SEM fatigue experiment system (JSM-5410V) at State Key Laboratory Coal Resources and Safe Mining at China University of Mining and Technology (Beijing) was employed in three-point-bending experiments, which consists of loading device, electron microscope, scanning equipment, loading control equipment, heating control equipment, and image acquisition equipment, as presented in Figure 1c. Before loading, the coal samples were plated with gold by SBC-12 small ion sputtering instrument (see Figure 1b) to improve electrical conductivity, and the gold-plating time was set to 110 s. The experimental temperature was set at 298 K. The processed coal sample was placed in the loading device with a preload of nearly 5 N, which could avoid falling off when the loading device was pushed into the viewing chamber. Simultaneously, the experimental data of load-displacement was recorded by load control equipment. The grayscale images for the propagation of micro crack on the samples were captured by scanning equipment. The displacement loading was adopted, and the loading head moves at a rate of 10^−4^ mm/s. The scanning voltage was set as 15 kV. The surface morphologies of the samples were obtained by secondary electronic imaging.

## 3. Experimental Results

### 3.1. Mercury Injection Porosimetry

**Figure 2:**
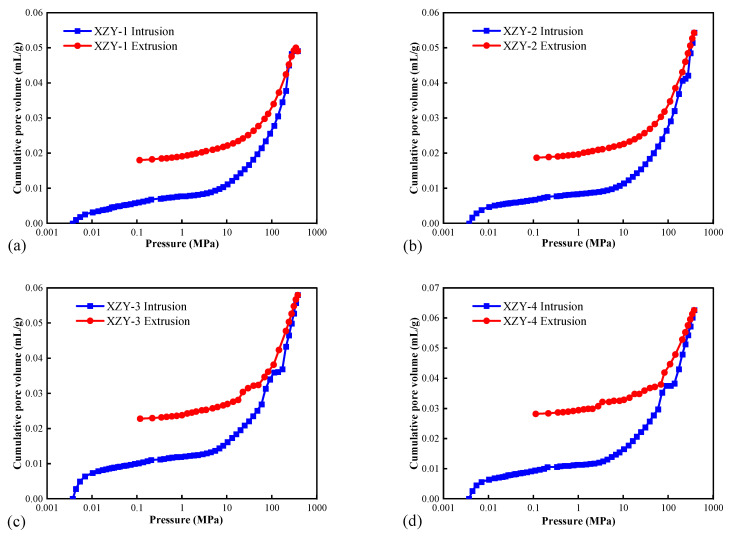
Mercury intrusion and extrusion curves of coal samples. (**a**) XZY-1; (**b**) XZY-2; (**c**) XZY-3; (**d**) XZY-4.

The pore volumes of XZY coal samples under the mercury injection pressure are illustrated in Figure 2. When the mercury injection pressure is less than 0.01 MPa, the cumulative pore volume increases sharply, and intergranular pores are filled quickly with mercury. When the pressure is up to 10 MPa, the isotherm grows linearly and mercury is injected into the pores whose diameter is approximately 120 nm. With the increment of the injection pressure, the volume increases rapidly again. Factors such as pore morphology, pore volume content, mercury injection pressure, and matrix compressibility lead to the difference in cumulative mercury injected volume [42].

After decreasing the intrusion pressure, mercury exits from the pores and micro-fractures. Due to the existence of opening pores, some mercury cannot exit promptly, or the exiting speed is slow, forming a hysteresis loop between the extrusion and injection curve. The width of the hysteresis loop can characterize the morphology of the pores and reveal the connectivity of pore network [9,43]. It can be seen from Figure 2 that hysteresis loops of XZY coal samples are wide, and the mean mercury withdrawal efficiency is 63.64%, implying that the pores are mainly opening pores with good pore connectivity.

### 3.2. Low-Temperature Nitrogen and Carbon Dioxide Gas Adsorption

The results from the gas adsorption experiments are listed in Table 1. The mean pore volume of the samples with diameter less than 2 nm is 0.0083 cm^3^/g acquired from LT-CA tests, which is higher than that results of LT-NGA. This may be as the coal samples in the LT-NGA experiment will shrink at the experimental temperature of 77 K, resulting in a decrease in pore volume; while in the LT-CA experiment, adsorption expansion occurred in the coal samples [16].

**Figure 3:**
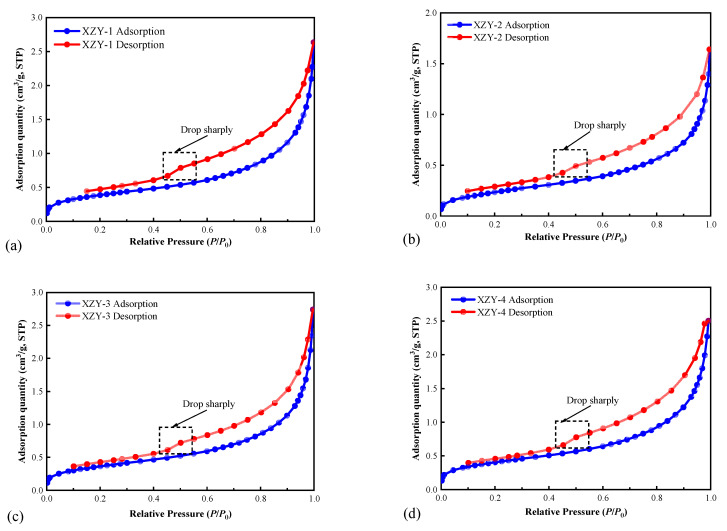
Low-temperature liquid N_2_ adsorption and desorption curves: (**a**) XZY-1; (**b**) XZY-2; (**c**) XZY-3; (**d**) XZY-4.

The results of LT-NGA are shown in Figure 3. The isotherm of the low-pressure stage (0 < *P*/*P*_0_ < 0.3) is close to the *P*/*P*_0_ axis, representing that the interaction force between coal matrix and nitrogen is weak. In the middle-pressure stage (0.3 < *P*/*P*_0_ < 0.8), due to the capillary condensation, N_2_ molecules condensed under pressure and filled the mesoporous channels of the coal sample. In the high-pressure stage (0.8 < *P*/*P*_0_ < 1.0), the pore volume increases sharply [6]. Adsorption saturation phenomenon is not observed when *P*/*P*_0_ is close to one, suggesting that the samples also have macro pores, which cannot be determined by LT-NGA experiments [44]. Hysteretic loop morphology reveals pore types. As shown in Figure 3, when *P*/*P*_0_ is lower both the desorption and adsorption curves are parallel, illustrating that the smaller pores are composed of both open and closed pores. When *P*/*P*_0_ is close to 0.5, the desorption curve drops rapidly, which means that there are numerous ink-bottle pores in this aperture segment and the corresponding pore connectivity is poor. When *P*/*P*_0_ is higher, the width of the hysteresis loop is larger, which suggests that the opening pores are in overwhelming superiority in the pores with a larger size.

### 3.3. Three-Point Bending Experiments

**Figure 4:**
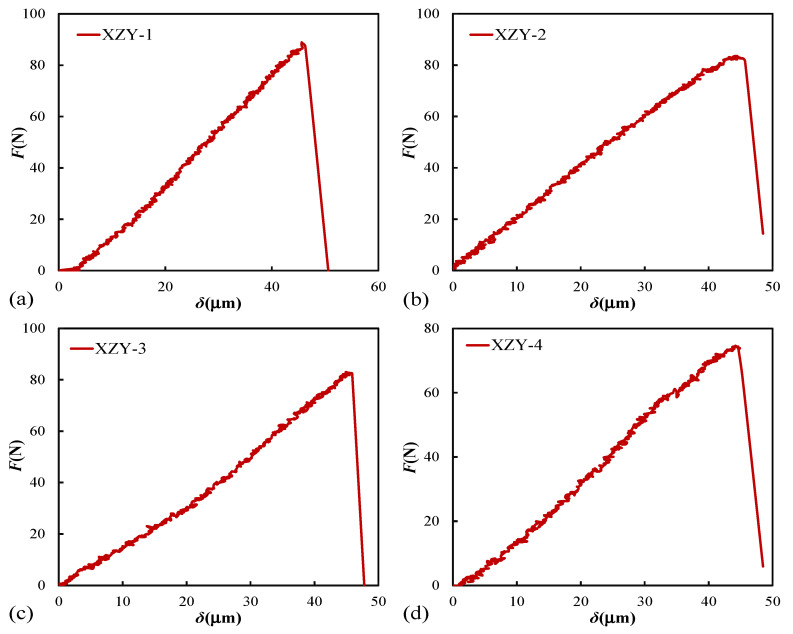
Load-displacement curves: (**a**) XZY-1; (**b**) XZY-2; (**c**) XZY-3; (**d**) XZY-4.

The curves of load-displacement for coal samples are displayed in Figure 4. Ignoring the clamping force of the fixture, the maximum extreme loading value is XZY-1 sample (88.9 N) and the minimum is XZY-4 sample (74.6 N). The average extreme loading value is 82.5 N. The fractured displacement of each coal sample is nearly equal, with an average value of 48.9 μm. On the load-displacement curves, it can be found that four specimens show obvious brittle failure characteristics during the period of rapid decline of bearing capacity after peak loading.

Bending elastic modulus reflects the elastic deformation capacity of a material. Tensile strength, estimated by a three-point bending experiment, can indirectly reveal the maximum capacity of a material to resist tensile damage. In the three-point bending experiment, the stress intensity factor determines the stress field and displacement field near the crack tip of the sample, and its critical value is named fracture toughness [45]. When the experimental temperature and loading rate keep stable, the fracture toughness is regarded as a constant, which is used as an index to judge the fracture stability of the material. The prefabricated notch of the coal sample is assumed as the initial crack, and the tensile stress at the notch is perpendicular to the crack plane. Therefore, the crack of coal samples is considered to be an opening crack (mode-I). For simple, virtual crack propagation is supposed to be zero. The depth of prefabricated notches affects the magnitude of peak load, and the fracture parameters of coal samples with prefabricated notches are calculated by Equations (1)–(3) [46,47].

(1) $$E_{b}=\frac{kl^{3}}{4b{\left(h-a\right)}^{3}}$$

(2) $$\sigma_{t}=\frac{3F_{\max}l}{2b{\left(h-a\right)}^{2}}$$

(3) $$K_{IC}=\frac{F_{\max}l}{bh^{1.5}}\left[2.9{\left(\frac{a}{h}\right)}^{0.5}-4.6{\left(\frac{a}{h}\right)}^{1.5}+21.8{\left(\frac{a}{h}\right)}^{2.5}-37.6{\left(\frac{a}{h}\right)}^{3.5}+38.7{\left(\frac{a}{h}\right)}^{4.5}\right]$$

where *E*_b_, *σ*_t_, and *K*_IC_ refer to the bending modulus (MPa), the tensile strength (MPa), and the fracture toughness (MPa·mm^1/2^), respectively. *k* represents slope of elastic stage in load-displacement curve. *a* denotes the length of prefabricated notch (mm). *h* and *b* stand for the height and thickness of the sample in mm, respectively. *l* is the effective support span (mm).

The fracture parameters of coal samples are listed in Table 2. The average tensile strength and bending modulus of coal samples are 7.73 MPa, and 1356 MPa, respectively. The mean fracture toughness of XZY coal samples is 12.195 MPa·mm^1/2^, suggesting that the XZY coal sample has strong fracture stability.

## 4. Discussion and Analysis

### 4.1. Multiscale Pore Size Distributions

When the mercury injection pressure is greater than 20 MPa in the MIP experiment, the coal matrix is compressed and the pore wall is destroyed under high mercury pressure [11,14], which is consistent with the references of Sun [12] and Li [13]. As a result, the pore volume measured is larger than the actual value. The cumulative pore volumes measured by MIP were modified by the gas adsorption methods to obtain accurate results.

Assume that mercury is incompressible, coal matrix compressibility can be defined as [14]:

(4) $$k_{c}=\frac{dV_{c}}{V_{c}dP}$$

where *k*_c_ is matrix compressibility coefficient in m^2^/N; *V*_c_ is the volume of coal matrix in mL/g and *P* is the mercury pressure in MPa. Since the coal sample contains partial micropores, which cannot be penetrated by mercury even at the maximal pressure, so *V*_c_ in Equation (4) also contains unfilled pores.

The change in coal matrix compression can be obtained by subtracting the pore filling increment (Δ*V_p_*) from the observed mercury volume increment (Δ*V_obs_*), that is:

(5) $$\Delta V_{c}=\Delta V_{obs}-\Delta V_{p}$$

Meanwhile, we assume that $\Delta V_{obs}/\Delta P$. is constant and replaced by *β* at the high mercury pressure range. Therefore, Δ*V_c_*/Δ*P* can be expressed as [15]:

(6) $$\frac{\Delta V_{c}}{\Delta P}=\frac{\Delta V_{obs}}{\Delta P}-\frac{\Delta V_{p}}{\Delta P}=\beta -\frac{\sum_{d_{\min}}^{d_{\max}}\Delta V_{p}}{\Delta P}$$

where *d*_min_ and *d*_max_ represent the minimal and maximal pore diameters in the compression range of coal matrix corresponding to higher mercury injection pressure, respectively. In this work, the mercury intrusion pressure that caused the coal matrix to be compressed is 20~413 MPa, and the corresponding pore diameter is 3~50 nm. The pore volume Δ*V*_p_ of the corresponding pore diameter is modified with LT-NGA data.

Substitute Equation (6) into Equation (4) and get the expression of *k*_c_:

(7) kc=ΔVcVcΔP=β−∑dmindmaxΔVpΔPVc

Using Equation (7), the compressibility coefficient is obtained, thus the accumulated pore volume before and after correction of the coal samples are determined, as listed in Table 3. The average of compressibility coefficient for XZY coal samples is 1.295 × 10^−10^ m^2^/N. The corrected porosity of XZY coal samples is 4.52% and the volume correction rates of the coal samples are 45.32%.

**Figure 5:**
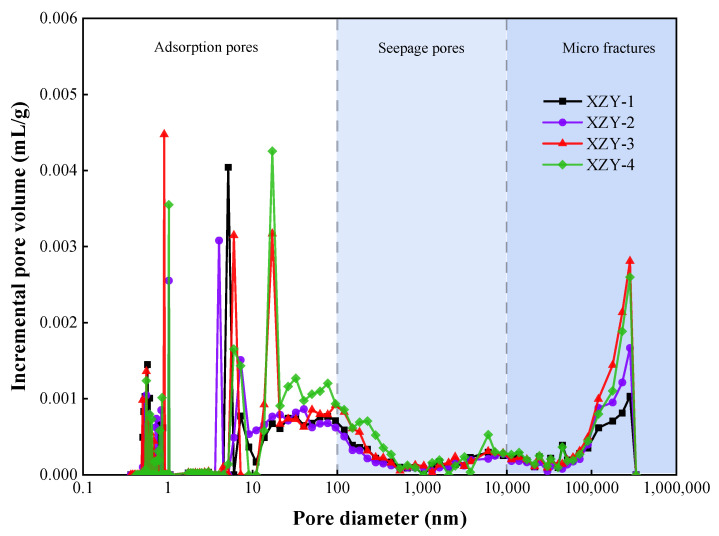
The pore size distributions of coal samples.

The PSDs of samples are shown in Figure 5. The PSDs of coal samples present multi-peak shapes. Thereinto, for adsorption pores (<100 nm), the PSDs exhibit a multimodal distribution with a main peak in the pore interval of 1~20 nm, which is convenient for the enrichment of gas. For pores size from 100 nm to 10,000 nm, there is a bimodal distribution, and the pore content is nearby zero in the valley points (500~2000 nm). The PSDs of micro fractures reveal a unimodal distribution with a peak in the pore size of 10~30 μm.

### 4.2. Surface Fractal Characteristics

Based on thermodynamic principles of porous materials, Zhang and Li proposed the relationship between the accumulated surface energy *W_n_* and the function of intrusion volume *Q_n_* during the mercury injection process [24]:

(8) $$\ln\left(W_{n}\right)=\ln\left(Q_{n}\right)+C$$

where

(9) $$W_{n}=\sum_{i=1}^{n}{\bar{P}}_{i}\Delta V_{i}$$

and

(10) $$Q_{n}=r_{n}^{2-D_{s}}V_{n}^{D_{s}/3}$$

where ${\bar{P}}_{i}$ is the mercury injected pressure at step *i*; Δ*V_i_* is the increment of mercury injected volume at pressure ${\bar{P}}_{i}$; *r_n_* is the smallest pore radius injected by mercury; *D_s_* is the surface fractal dimension; *C* is a constant.

**Figure 6:**
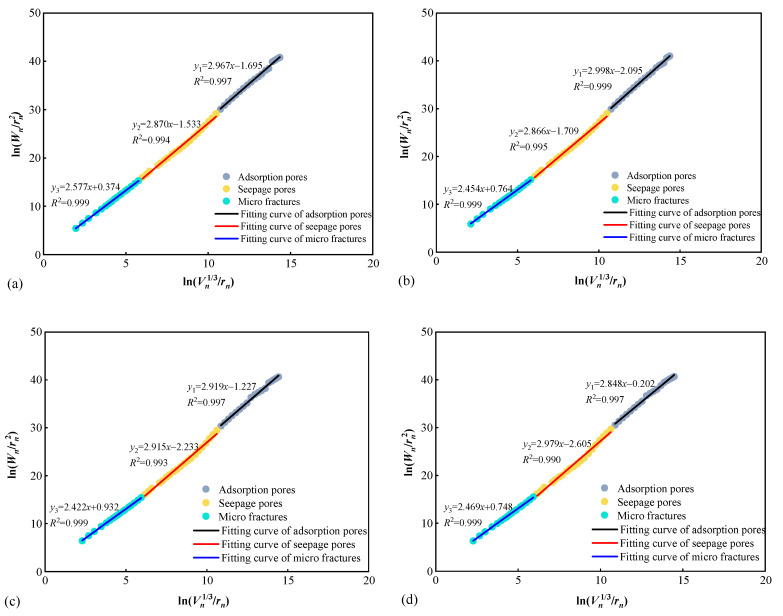
Surface fractal dimensions of coal samples. (**a**) XZY-1; (**b**) XZY-2; (**c**) XZY-3; (**d**) XZY-4.

The surface fractal dimension *D_s_* is evaluated by the slope of the curve $\ln\left(W_{n}/r_{n}^{2}\right)$ vs. $\ln\left(V_{n}^{1/3}/r_{n}\right)$ in Figure 6, that is [23]:

(11) $$D_{s}=\frac{d\ln\left(W_{n}/r_{n}^{2}\right)}{d\ln\left(V_{n}^{1/3}/r_{n}\right)}$$

As shown in Figure 6, the surface fractal dimension of coal is scale-dependent, and this characteristic has been proved in other porous materials [48,49]. The three pore-size segments correspond to adsorption pore fractal range *D*_s1_ (*d* < 100 nm), seepage pore fractal range *D*_s2_ (100 nm< *d* < 10,000 nm), and micro fracture fractal range *D*_s3_ (*d* > 10,000 nm). The pores in the three size ranges have fractal characteristics. The surface fractal results for XZY coal samples are different, as displayed in Table 4.

As can be seen from Table 4, the average surface fractal dimension of adsorption pores is 2.933, and is 0.025 and 0.452 larger than that of seepage pores and micro-fractures, respectively, indicating that the pore surfaces roughness of three pore regions are various. The adsorption pores have the largest volume, and smallest pore size and widest surface area. Therefore, the fractal dimension of adsorption pore is the largest. The pore size of micro-cracks is larger than the seepage pores, while the pore surface is relatively smooth, resulting in smallest surface fractal dimension. The content of seepage pores is 0.0048 mL/g. The surface fractal dimension mean value of seepage pores is between that of adsorption pores and micro fracture, indicating that the roughness of seepage pores is not too complex, which is beneficial to gas seepage or gas extraction.

### 4.3. Microcrack Propagation Process

**Figure 7:**
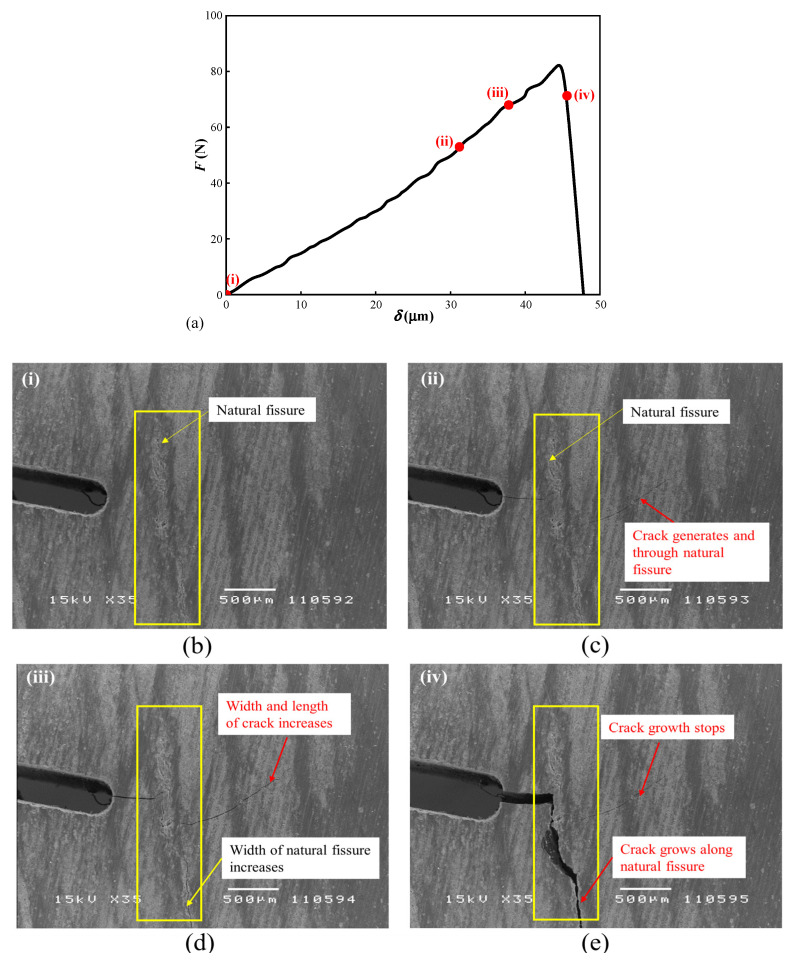
Micro-crack morphology on XZY-3 sample under different loads. (**a**) The load-displacement curve; (**b**) Before load; (**c**) Micro crack is spotted; (**d**) Micro crack propagation; (**e**) The width of main crack formed.

The surfaces of coal samples were scanned synchronously by SEM to observe micro crack generation and development in the process of loading. Pictures of the cracks at the prefabricated notch of the XZY-3 coal sample are shown in Figure 7b–e at the instances (I–IV) marked on the load–displacement curve in Figure 7a. At the beginning of loading, there is no crack at the precast notch, but there is a natural fissure near the notch (see Figure 7b). When the load increases to 53.6 N, the micro crack is spotted at the tip of the fabricated notch and through the natural fissure (see Figure 7c). When the load reaches 68 N, the width of the micro crack and the opening of the natural fissure increases, and the displacement of the corresponding load head is 37.8 μm (see Figure 7d). Due to the SEM scan interval, all scanning images of crack propagation during the fracture process cannot be taken, e.g., crack morphology corresponding to peak load. After the peak load, the load-bearing capacity of the sample decreases, while the displacement continues to raise, and the main crack expands along the direction of the natural crack (see Figure 7e). Finally, the sample loses the load-bearing ability, and the corresponding displacement reaches 47.8 μm.

**Figure 8:**
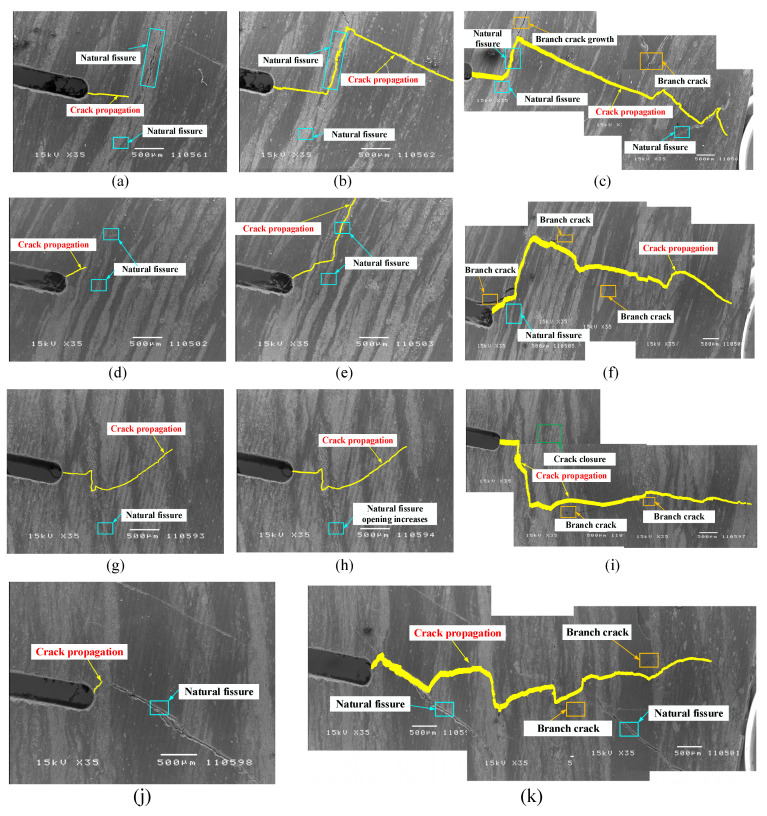
Crack propagation path under different loads. (**a**) XZY-1 (63.8 N); (**b**) XZY-1 (73.4 N); (**c**) XZY-1 (fractured); (**d**) XZY-2 (59.8 N); (**e**) XZY-2 (78.1 N); (**f**) XZY-2 (fractured); (**g**) XZY-3 (53.6 N); (**h**) XZY-3 (67.7 N); (**i**) XZY-3 (fractured); (**j**) XZY-4 (53.5 N); (**k**) XZY-4 (fractured).

Coal is a heterogeneous material, which comprises mineral particles with different mechanical properties, such as amorphous substance, dolomite, clay minerals, calcite, etc. [50]. According to the principle of minimum energy consumption, the main crack of coal sample expands along the path that consumes the lowest energy under the action of external force [51]. Cracks develop along with the mineral interface, resulting in the cracks in a bent state, rather than through the development of minerals, which may consume more energy. The SEM images of the crack propagation path on the coal sample under different loads are shown in Figure 8, and the path of the main crack is indicated by yellow pixels. The main crack starts from the prefabricated notch, and does not always propagate in the direction of vertical tension. Deflection of the direction of crack propagation may be related to the weak structure. The process of coal sample deformation and failure is accompanied by the development and evolution of spatial morphology, which is a process of micro cracks initiation, development, and penetration. During the initial stage of loading, many natural pores and cracks near the prefabricated notch tip are compressed. With the further increase in the load, the microscopic cracks forms, expands, merges, and develops into macroscopic cracks, which are visible to the naked eye. Finally, the sample has no load-bearing capacity and macroscopic fracture occurs. Except for the main cracks, there are many branch cracks on the failure samples. The high occurrence area of branch cracks is generally the place where the growth rate of the main crack slows down [33].

The size parameters and fractal dimensions of main cracks are listed in Table 5. The length and opening of the main cracks after fracture are measured. Thereinto, the linear length of the crack refers to the length of the line connecting the beginning and the end of the crack, and the curve length of crack refers to the length of the curve after being extracted and refined. It can be seen from Table 5 that the curve lengths of the cracks fractured are larger than the linear lengths, which demonstrates that the degree of bending in the process of crack growth is large.

Although the morphology of a crack is random and irregular during crack propagation, it is self-similar and can be characterized by fractal dimensions [52,53]. Surface fractal dimension *D* of the main crack indicates the bending degree and distribution density of the crack, and can also reveal the damage degree of porous materials.

The box counting method, widely used in the calculation of fractal dimension as a result of its automaticity [54], is applied to illustrate the fractal feature of crack propagation route in this work. Assume that the size length of the box is *ε_i_*, and the number of images covered by the box is *N*(*ε_i_*). The value of *D* is expressed as [55]:

(12) $$D=\underset{\varepsilon \to 0}{\lim}\frac{\log\left(N_{\varepsilon i}\right)}{\log\left(1/\varepsilon i\right)}$$

The fractal dimensions of main cracks are listed in Table 5. The process of crack propagation is also the process of increasing fractal dimension. The mean fractal dimension of the crack path on coal samples after fracture is 1.445. Although the peak load of the XZY-4 sample is the lowest, the final fractal dimension of the crack path on the XZY-4 sample is the largest, suggesting that the crack propagation path of the XZY-4 sample is the most complex.

### 4.4. Fracture Energy Analysis

Fracture energy is the energy required to expand the crack length per unit area. It is also one of the important fracture mechanics parameters of materials, similar to fracture toughness [56,57]. It is assumed that all the work carried out by external forces is used for crack propagation [58]. For three-point bending experiment, the work carried out by the load head is equal to the area formed by the load-displacement curve and the two coordinate axes. As the gravity direction is perpendicular to the loading direction, the gravitational potential energy is ignored. The fracture energy is expressed as:

(13) $$G_{f}=\frac{\int_{0}^{\delta_{e}}Fd\delta }{\left(h-a\right)b}$$

Here, *G*_f_ stands for fracture energy in N/mm; *δ*_e_ denotes the displacement at the end of the loading in mm; $\int_{0}^{\delta_{e}}Fd\delta$ represents the area enclosed under the load-displacement curve in N·mm.

**Figure 9:**
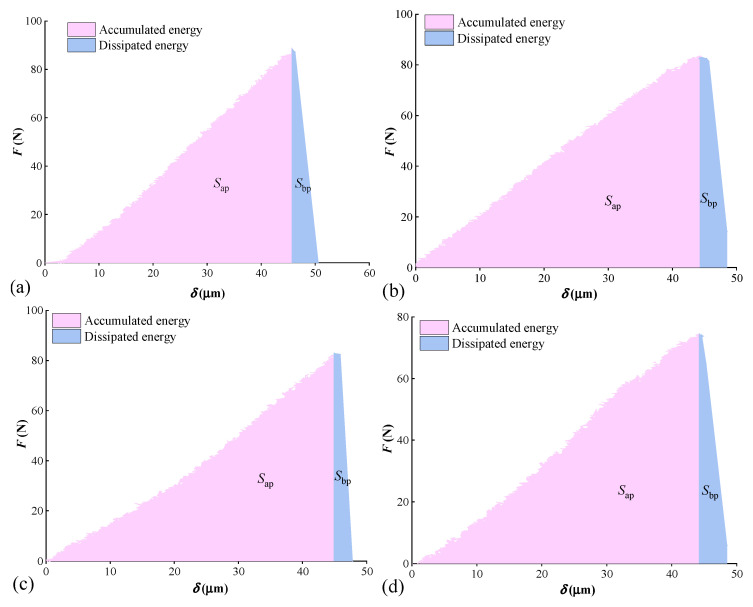
The energy accumulated and dissipated from coal samples: (**a**) XZY-1; (**b**) XZY-2; (**c**) XZY-3; (**d**) XZY-4.

Three-point bending fracture process is a process of energy accumulation and dissipation. When the load increases from zero to maximum, the work carried out by load is considered as an accumulated energy, which is manifested as the expansion of micro cracks. The accumulated energy is equal to the area (*S*_bp_) surrounded by the load and displacement of the pre-peak stage, as shown in Figure 9. After the load reaches the peak, the accumulated energy begins to dissipate, which is represented by a failure of coal samples. The dissipated energy is equal to the area (*S*_ap_) enclosed by load and displacement of the post-peak stage, as shown in Figure 9. The ratio of the dissipated energy (*S*_ap_) to the accumulated energy (*S*_bp_) is expressed by the energy accumulation dissipation index *α*, that is [33]:

(14) $$\alpha =\frac{S_{ap}}{S_{bp}}$$

The smaller the *α* value is, the more brittle the material is. Otherwise, the ductility of the material is better. In addition, according to the definition of rock failure time [59], the bending failure time is defined as the time from peak load to failure in three-point bending. The shorter the bending failure time is, the more brittle the coal sample is. The fracture energy and bending fracture time of coal samples are shown in Table 6.

From Table 6, the average fracture energy of the XZY samples is 0.049 N/mm, but the accumulated energy and dissipated energy of samples are 1.77 N∙mm and 0.21 N∙mm, respectively. It indicates that the pre-peak energy of the sample is large, while the post-peak energy release is small. The value of *α* suggests that the difference between the accumulated energy and dissipated energy index of the XZY coal sample is great, and the brittle fracture occurs in samples. It can be verified by the extremely short failure time after peak load.

## 5. Conclusions

(1) The characteristics for coal samples with pore sizes ranging less than 370 μm was investigated using MIP, LT-NGA and LT-CA experiments. Meanwhile, the pore types of XZY coal samples are mainly opening pores. The coal matrix compression makes the measured pore volume larger, when mercury injection pressure greater than 20 MPa. The matrix compressibility coefficient is in the range of 1.278~1.309 × 10^−10^ m^2^/N. Removing the matrix compressibility effect, the mean corrected pore volume is 0.0352 mL/g.
(2) The PSDs of XZY coal samples have multi-peak shapes. The adsorbed pore volume with an average of 0.0224 mL/g is the largest, followed by micro fractures. The volume content of seepage pores with sizes ranging from 100 nm to 10,000 nm accounts for 13.22% of the total volume. The surface fractal dimensions of adsorption pores, seepage pores, and micro fractures decrease in sequence, indicating that the pore structure is convenient for gas seepage, which explain the reason for high gas content in the 14-3# coal seam, but there was almost no gas outburst accident so far.
(3) Based on the three-point bending experiment, the propagation path of the main crack is mainly affected by the natural crack and loading. With the increase in load, the main crack tends to expand almost perpendicular to the tensile direction until the specimen fails. Meanwhile, the fractal dimension of the main crack path increases, which changes from one to two. Both the energy accumulation and dissipation index *α* and bending failure time reveal the brittle failure characteristics of XZY coal samples.

## Figures and Tables

**Table 1:** Gas adsorption experimental results.

Sample No.		Parameters Acquired from LT-CA Experiments			Parameters Acquired from LT-NGA Experiments					
	Minimum Pore Diameter (nm)	Maximum Pore Diameter (nm)	Surface Area (cm^2^/g)	Total Pore Volume (cm^3^/g)	BET Average Pore Diameter (nm)	BET Surface Area (cm^2^/g)	Micro Pore Volume (cm^3^/g)	Transition Pore Volume (cm^3^/g)	Meso Pore Volume (cm^3^/g)	Total Pore Volume (cm^3^/g)
XZY-1	0.3669	1.0657	23.49	0.0072	11.821	1.380	0.00086	0.00184	0.00069	0.0034
XZY-2	0.3669	1.0657	22.28	0.0081	11.551	0.879	0.00063	0.00109	0.00043	0.0022
XZY-3	0.3669	1.0657	24.68	0.0088	12.856	1.319	0.00087	0.00188	0.00080	0.0036
XZY-4	0.4193	1.0657	22.54	0.0089	12.622	1.447	0.00091	0.00201	0.00084	0.0038
Average	0.3800	1.0657	23.25	0.0083	12.213	1.256	0.00082	0.00171	0.00069	0.0032

**Table 2:** The fracture parameters of coal samples.

Sample No.	*F*_max_ (N)	*δ*_max_ (μm)	*k* (N/μm)	*E*_b_ (MPa)	*σ*_t_ (MPa)	*K*_IC_ (MPa·mm^1/2^)
XZY-1	88.9	50.6	1.81	1413	8.33	13.141
XZY-2	83.5	48.5	2.01	1569	7.83	12.343
XZY-3	83.0	47.8	1.51	1177	7.78	12.269
XZY-4	74.6	48.5	1.62	1263	6.99	11.027
Average	82.5	48.9	1.74	1356	7.73	12.195

**Table 3:** Pore structure parameters before and after compressibility correction.

Sample No.	*β* (10^−5^)	*k*_c_ (10^−10^ m^2^/N)	Porosity Before Correction (%)	Porosity after Correction (%)	Accumulated Pore Volume before Correction (mL/g)	Accumulated Pore Volume after Correction (mL/g)	Volume Correction Rate (%)
XZY-1	10.8	1.278	6.32	3.85	0.0565	0.0299	47.08
XZY-2	10.8	1.309	6.95	4.11	0.0625	0.0321	48.64
XZY-3	10.4	1.293	7.45	4.83	0.0670	0.0376	43.88
XZY-4	11.0	1.299	8.04	5.31	0.0717	0.0413	42.40
Average	10.8	1.295	7.19	4.52	0.0644	0.0352	45.32

**Table 4:** The results of pore fractal dimensions of coal samples.

Sample No.	*D*_s1_	*D*_s2_	*D*_s3_	*V*_a_ (mL/g)	*V*_s_ (mL/g)	*V*_f_ (mL/g)
XZY-1	2.967	2.870	2.577	0.0197	0.0042	0.0060
XZY-2	2.998	2.866	2.454	0.0217	0.0036	0.0068
XZY-3	2.919	2.915	2.422	0.0225	0.0050	0.0101
XZY-4	2.848	2.979	2.469	0.0257	0.0062	0.0094
Average	2.933	2.908	2.481	0.0224	0.0048	0.0081

Note: *V*_a_ represents adsorption pore volume; *V*_s_ represents seepage pore volume; *V*_f_ represents micro-fracture.

**Table 5:** The size parameters and fractal dimensions of main cracks during loading.

Sample No.	The Stage before Peak Load					The Stage after Peak Load				
	Load 1 (N)	Crack Initial Spreading Angle (°)	Fractal Dimension	Load 2 (N)	Fractal Dimension	Load 3 (N)	Crack Curve Length (mm)	Crack Linear Length (mm)	Maximum Crack Opening (mm)	Fractal Dimension
XZY-1	63.8	79	1.285	73.4	1.292	43.6	9.47	7.30	0.118	1.362
XZY-2	59.8	48	1.207	78.1	1.253	14.4	8.94	7.38	0.101	1.429
XZY-3	53.6	84	1.215	68	1.237	71.3	8.77	7.28	0.112	1.433
XZY-4	53.5	31	1.218	/	/	64.7	8.53	7.13	0.096	1.557

**Table 6:** Fracture energy parameters and bending fracture time of the coal samples.

Sample No.	*G*_f_ (N/mm)	Accumulated Energy (N∙mm)	Time before Peak Load (s)	Dissipated Energy (N∙mm)	Time after Peak Load (s)	*α*
XZY-1	0.052	1.816	592	0.249	5	0.14
XZY-2	0.055	1.982	570	0.233	12	0.12
XZY-3	0.046	1.684	600	0.161	5	0.10
XZY-4	0.045	1.600	573	0.191	11	0.12
Average	0.049	1.77	583	0.21	8	0.12

## Data Availability

The raw data required to reproduce these results cannot be shared at this time as the data also form part of an ongoing study.

## List of Markings

*E*_b_	bending modulus, MPa
*σ*_t_	tensile strength, MPa
*K*_IC_	fracture toughness, MPa·mm^1/2^
*k*	slope of elastic stage
*a*	length of prefabricated notch, mm
*h*	height of the sample, mm
*b*	thickness of the sample, mm
*l*	effective support span, mm
*k*_c_	matrix compressibility coefficient, m^2^/N
*V*_c_	volume of coal matrix, mL/g
*P*	mercury pressure, MPa
Δ*V_obs_*	observed mercury volume increment, mL/g
*β*	constant
*d*_min_	minimal pore diameters, nm
*d*_max_	maximal pore diameters, nm
*W_n_*	accumulated surface energy
*Q_n_*	function of intrusion volume
${\bar{P}}_{i}$	mercury injected pressure at step *i*, MPa
Δ*V_i_*	increment of mercury injected volume at pressure ${\bar{P}}_{i}$, mL/g
*r_n_*	smallest pore radius injected by mercury, nm
*D*_s_	surface dimension
*C*	constant
*ε_i_*	size of the box
*N*(*ε_i_*)	number of images covered by the box
*G*_f_	stands for fracture energy, N/mm
*δ*_e_	displacement at the end of the loading, mm
*α*	energy accumulation dissipation index
